# The Diagnostic Capability of Swept Source OCT Angiography in Treatment-Naive Exudative Neovascular Age-Related Macular Degeneration

**DOI:** 10.1155/2021/6695918

**Published:** 2021-02-16

**Authors:** Daniel Ahmed, Martin Stattin, Anna-Maria Haas, Stefan Kickinger, Maximilian Gabriel, Alexandra Graf, Katharina Krepler, Siamak Ansari-Shahrezaei

**Affiliations:** ^1^Karl Landsteiner Institute for Retinal Research and Imaging, Rudolf Foundation Hospital, Juchgasse 25, Vienna 1030, Austria; ^2^Department of Ophthalmology, Rudolf Foundation Hospital, Juchgasse 25, Vienna 1030, Austria; ^3^Department of Ophthalmology, Medical University of Graz, Auenbruggerplatz 1, Graz 8036, Austria; ^4^Center for Medical Statistic Informatics and Intelligent Systems, Medical University of Vienna, Spitalgasse 23, Vienna 1090, Austria

## Abstract

**Purpose:**

To evaluate the capability of swept source-optical coherence tomography angiography (SS-OCTA) in the detection and localization of treatment-naive macular neovascularization (MNV) secondary to exudative neovascular age-related macular degeneration (nAMD).

**Methods:**

In this prospective, observational case series, 158 eyes of 142 patients were diagnosed with exudative nAMD using fluorescein (FA) and indocyanine green angiography (ICGA) and evaluated by SS-OCTA in a tertiary retina center (Rudolf Foundation Hospital Vienna, Austria). The main outcome measure was the sensitivity of SS-OCTA compared to the standard multimodal imaging approach. Secondary outcome measure was the anatomic analysis of MNV in relation to the retinal pigment epithelium.

**Results:**

En-face SS-OCTA confirmed a MNV in 126 eyes (sensitivity: 79.8%), leaving 32 eyes (20.2%) undetected. In 23 of these 32 eyes (71.9%), abnormal flow in cross-sectional SS-OCTA B-scans was identified, giving an overall SS-OCTA sensitivity of 94.3%. Eyes with a pigment epithelium detachment (PED) ≥ 300 *μ*m had a smaller probability for correct MNV detection (*p*=0.015). Type 1 MNV showed a trend (*p*=0.051) towards smaller probability for the correct detection compared to all other subtypes. Other relevant factors for the nondetection of MNV in SS-OCTA were image artifacts present in 3 of 32 eyes (9.4%). SS-OCTA confirmed the anatomic localization of 93 in 126 MNVs as compared to FA (sensitivity: 73.8%). There was no influence of age, gender, pseudophakia, visual acuity, central foveal thickness, or subfoveal choroidal thickness on the detection rate of MNV.

**Conclusions:**

SS-OCTA remains inferior to dye-based angiography in the detection rate of exudative nAMD consistent with type 1 MNV and a PED ≥300 *µ*m. The capability to combine imaging modalities and distinguish the respective MNV subtype improves its diagnostic value.

## 1. Introduction

Dilated fundus examination and multimodal imaging including optical coherence tomography (OCT), fluorescein angiography (FA), and indocyanine green angiography (ICGA) are currently utilized to diagnose neovascular age-related macular degeneration (nAMD) [1–3]. Type 1 macular neovascularization (MNV) and polypoidal type 1 MNV remain under the retinal pigment epithelium (RPE), whereas type 2 MNV is located in the subretinal space [4, 5]. A mixed type MNV is composed of new vessels growing in more than one layer. Both, type 1 and type 2 MNV originate from the choroid, while type 3, formerly described as retinal angiomatous proliferation, reflects a distinct form of nAMD with intraretinal neovascularization and possible involvement of the choroid [6, 7]. MNV was proposed as a more suitable term to sum up all types of nAMD in contrast to choroidal neovascularization (CNV) [8, 9].

OCT angiography (OCTA) has emerged as a fast and noninvasive imaging technique to evaluate retinal pathologies [10, 11]. OCTA's dye-less visualization of retinal vasculature is based on changes in intensity or phase contrast between multiple repeated OCT B-scans, caused by moving blood cells [12, 13]. The recent implementation of OCTA in clinical routine enabled new possibilities for the detection, analyzation, and observation of MNV in AMD [14–16]. At the moment, commercially available for a few years, swept source-OCTA (SS-OCTA) is the most promising OCTA technology with a longer wavelength and greater signal-to-noise ratio at greater imaging depths, resulting in deeper tissue penetration, better visualization of microvascular structures, and higher diagnostic accuracy compared to spectral domain-OCTA (SD-OCTA) [17–21]. The comparison of MNV area size in different imaging techniques including ICGA, SD-OCTA, and SS-OCTA remains challenging, although SS-OCTA tends to correlate better with ICGA in most recent studies [22–24]. Our study group retrospectively investigated the detection rate of nAMD using SS-OCTA, giving a sensitivity of 75.7% in 107 eyes (98 patients) [25]. As a consequence, the aim of this study was a verification of our data in a prospective manner. Again, dye-based angiography was used for detecting and subtyping nAMD as standard of care as opposed to an analysis solely based on SS-OCTA in a representative number of eyes. Additionally, MNV subtypes were classified by means of SS-OCTA.

## 2. Methods

A prospective masked noninferiority study design to compare the standard multimodal imaging approach to SS-OCTA alone in a clinical setting. The study was approved by the Viennese ethics committee (EK-17-083-0517) and performed in accordance with the tenets of the Declaration of Helsinki.

### 2.1. Study Population

This observational case series included consecutive patients with the clinical picture of exudative nAMD, referred to our tertiary center (Medical Retina Unit, Department of Ophthalmology, Rudolf Foundation Hospital Vienna, Austria; Karl Landsteiner Institute for Retinal Research and Imaging) for the detection and classification of MNV between August 2017 and January 2019. All patients routinely underwent a complete ophthalmic examination including indirect slit-lamp biomicroscopy (Haag-Streit AG, Bern, Switzerland) with dilated pupils using 0.5% tropicamide (Mydriaticum®, Agepha Pharmaceuticals, Vienna, Austria) and multimodal imaging with spectral domain- (SD-) OCT, FA, and ICGA (SPECTRALIS HRA-OCT Confocal Scanning Laser Ophthalmoscope and Angiography; Heidelberg Engineering, Heidelberg, Germany) as baseline standard of care. Best corrected visual acuity (BCVA) was measured using the Early Treatment Diabetic Retinopathy Study (ETDRS) letter score (4m) and converted to Snellen (Sn). Exclusion criteria were previous invasive ocular treatment for nAMD at any time.

### 2.2. Swept Source Optical Coherence Tomography Angiography Protocol

All investigated patients were additionally examined by a SS-OCTA device (DRI OCT Triton Plus; Topcon Corporation, Tokyo, Japan) on the same day of the first consultation. This device works at a center wavelength of 1050 nm under acquisition of 100,000 A-scans per second with a motion contrast algorithm called OCTARA™ [26]. Two trained operators captured 4.5 × 4.5 mm, 6 × 6mm and 9 × 9 mm OCTA macular cubes with a scan resolution of 320 × 320 (4.5 × 4.5 mm) or 512 × 512 (6 × 6 mm and 9 × 9 mm) SS-OCT B-line scans for each eye. The device operates on 1 mW input power with a digital axial resolution of 2.6 *µ*m and a transverse digital resolution range from 9.4 to 18.8 *µ*m depending on the selected cube.

### 2.3. Image Grading

The diagnosis of AMD including the classification of MNV into types 1–3, mixed types 1 and 2, or polypoidal type 1 was evaluated by two medical retina experts based on the multimodal imaging approach. Two independent graders, who were masked to angiographic findings, utilized all available en-face SS-OCTA images for grading in a binary manner (0 = MNV absent, 1 = MNV present). The integrated OCTA-analysis software IMAGEnet 6 (Version 1.24.1.15742, Topcon Corporation, Tokyo, Japan) was used to alter the corresponding automated OCT segmentation lines in the choriocapillaris or outer retina slab according to the MNV position. In the case of MNV presence in en-face SS-OCTA, the localization was determined in relation to the RPE (0 = MNV under the RPE, 1 = MNV above the RPE, 2 = MNV under and above the RPE, and 3 = MNV position not attributable). The maximum height of PED was measured in SS-OCT B-scans as the distance between Bruch's membrane and the maximum of the outer border of the RPE detachment. The central foveal thickness (CFT) was measured between Bruch's membrane and the umbo of the fovea. Subfoveal choroidal thickness (SFCT) was defined as the greatest vertical distance between Bruch's membrane and the sclerochoroidal interface. In case of severe segmentation artifacts, resegmentation was performed either semiautomated by altering the predefined sections or by drawing the lines manually. The automated artifact removal tool was used optionally; however, no further image postprocessing was performed for quality enhancement. Color-coded cross-sectional SS-OCTA B-scans were analyzed for abnormal flow in eyes with MNV absence in en-face picture. In the case of PED presence, cross-sectional SS-OCTA B-scans were used to display the corresponding en-face SS-OCTA segmentation as suggested by Tan et al. [27]. All eyes with MNV absence in en-face SS-OCTA were evaluated for relevant image artifacts possibly obscuring the MNV visualization. In case of grading disagreement, the images were independently re-evaluated. A senior clinical advisor was consulted if disagreement persisted.

### 2.4. Statistics

Sensitivity of SS-OCTA was calculated for neovascular AMD as defined by FA and ICGA. Age, gender, pseudophakia, BCVA, CFT, SFCT, PED, and MNV subtypes were investigated as potential factors influencing the detection rate using generalized mixed effects models with random factor patient. To investigate the predictive ability of PED, ROC-curves and the Youden index were applied to determine a possible cut-off value. *p* values smaller than 0.05 were considered as statistically significant. Intergrader variability was calculated using Cohen's kappa coefficients and corresponding confidence intervals. All analyses were performed using *R*, release 3.3.3, and SAS 9.4. system (SAS Institute Incorporated, Cary, USA). Tables were illustrated by using Microsoft Excel 2019 (Microsoft Corporation, Redmond, USA). Figures were composed utilizing Photoshop CC 14.0 (Adobe Systems Incorporated, San Jose, USA).

## 3. Results

Overall, slit-lamp biomicroscopy combined with SD-OCT, FA, and ICGA led to the diagnosis of treatment-naive exudative nAMD in 158 eyes of 142 patients. The demographics of the patients are displayed in Table 1.

**Figure 1:**
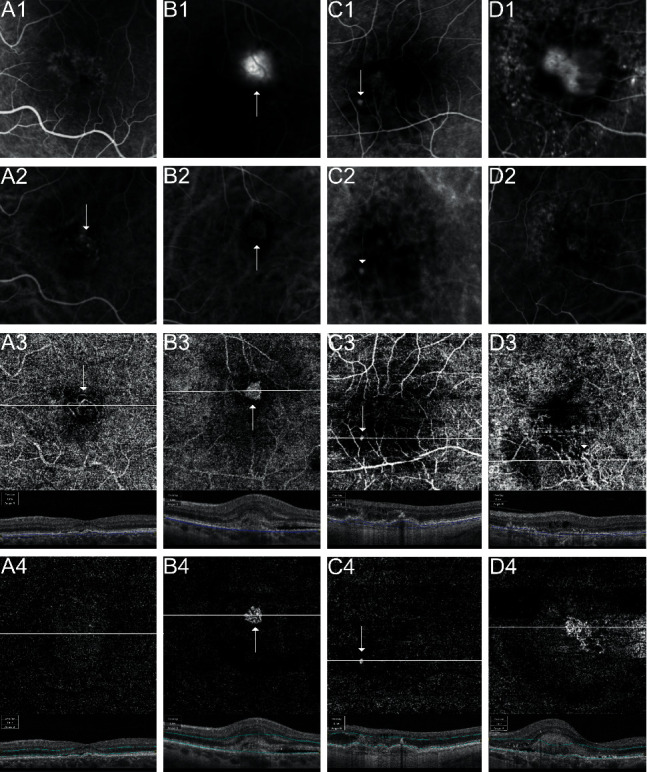
Detection of macular neovascularization (MNV) in age-related macular degeneration (AMD) by fluorescein angiography (FA), indocyanine green angiography (ICGA), and 4.5 × 4.5 mm en-face swept source; optical coherence tomography angiography (SS-OCTA). (A1) Type 1 MNV with speckled hyperfluorescence in FA (A2) ICGA visualized the neovascular lesion (arrow). (A3) Evidence of MNV (arrow) in en-face SS-OCTA with choriocapillaris (CC) segmentation under the retinal pigment epithelium (RPE) but (A4) absence of flow in the outer retina (OR) slab. (B1) Leakage in FA consistent with a type 2 MNV (arrow) in a left eye. (B2) Early ICGA highlighted a well demarcated MNV (arrow). (B3) Automated en-face SS-OCTA CC scan with a dense MNV (arrow) surrounded by a dark halo and projection artifacts of the superficial retinal vessels. (B4) Automated en-face SS-OCTA OR segmentation with the same neovascular complex in an otherwise nonvascularized tissue. (C1) A hyperfluorescent spot (arrow) in early FA and (C2) late ICGA diagnosed as type 3 MNV. (C3) Evidence of a neovascularization in the en-face SS-OCTA with CC and (C4) OR segmentation. (D1) Mixed type MNV with early leakage surrounded by speckled hyperfluorescence in FA (D2) ICGA with a neovascular lesion. (D3) MNV presence (circle) under the RPE in the en-face SS-OCTA CC slab and (D4) clear evidence of MNV over the RPE in the OR segmentation.

En-face SS-OCTA was able to depict a MNV formation in 126 eyes (Sensitivity: 79.8%), while no MNV could be detected in 32 eyes (20.2%) (Table 2; Figure 1).

**Figure 2:**
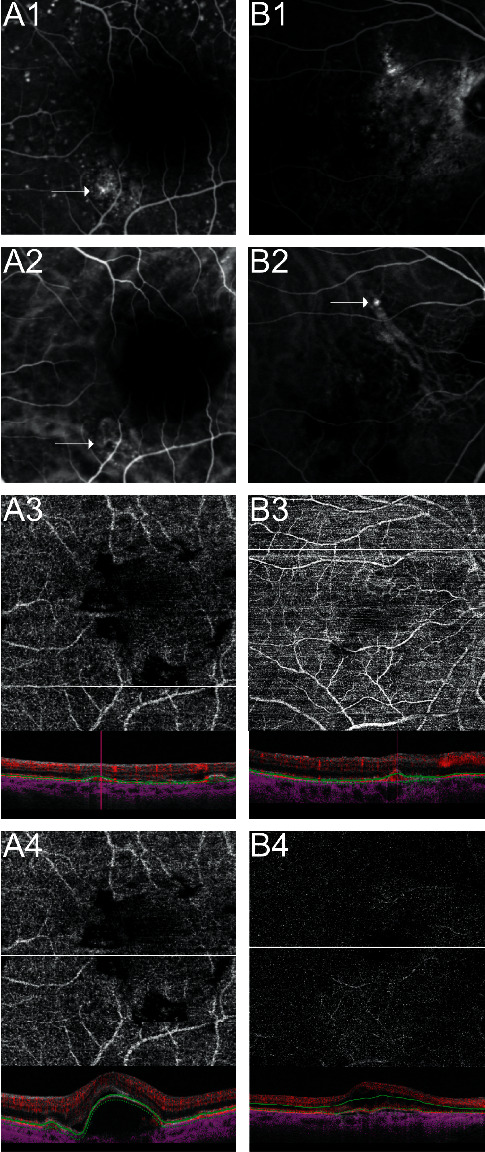
Detection of macular neovascularization (MNV) in age-related macular degeneration (AMD) by fluorescein angiography (FA), indocyanine green angiography (ICGA), and cross-sectional swept source-optical coherence tomography angiography (SS-OCTA). (A1) Early FA with drusen staining and a focal speckled hyperfluorescence (arrow) representing type 1 MNV in a left eye. (A2) Early ICGA with a circumscribed neovascular lesion (arrow) at the same location as shown in FA. (A3) Manual segmentation of a 4.5 × 4.5 mm en-face OCTA CC slab without evidence of a MNV membrane below the retinal pigment epithelium (RPE) besides projection artifacts and signal loss but flow density in color coded cross-sectional SS-OCTA. (A4) Manual segmentation of a 4.5 × 4.5 mm en-face OCTA CC slab across the fovea and cross-sectional SS-OCTA with no flow suspicious of a neovascularization in an otherwise dome-shaped pigment epithelium detachment. (B1) Ill-defined hyperfluorescence and drusen staining in early FA. (B2) ICGA revealed a hypercyanescent nodule consistent with a polypoidal lesion corresponding to (B3) cross-sectional flow density in SS-OCTA CC slab through the point of interest. (B4) 9 × 9 mm en-face OCTA OR slab at the fovea with hollow cystoid spaces besides projection artifacts but no evidence of neovascularization.

In 23 of these 32 eyes (71.9%), cross-sectional SS-OCTA B-scans revealed abnormal flow patterns in the respective segmentations (Figure 2).

The combination of en-face and cross-sectional OCTA confirmed a MNV in 149 of 158 eyes (sensitivity: 94.3%). Good intergrader agreement was observed initially (Κ = 0.91, CI = 0.87–0.96), increasing after independent reevaluation of the questionable findings (*K* = 0.98, CI = 0.95–1). The topographic localization defined by SS-OCTA was consistent with multimodal imaging in 93 of 126 eyes (sensitivity: 73.8%) (Table 3).

Intergrader agreement was lower for the topographical analysis of MNV formations regardless of the re-evaluation process (*K* = 0.76, CI = 0.65–0.87; *K* = 0.88, CI = 0.79–0.96).

Type 1 MNV showed a trend (*p*=0.051) towards smaller probability for the correct detection compared to all other subtypes. To investigate the predictive ability of MNV in a PED, we further plotted the receiver operating characteristic (ROC) curve. Overall, PED height showed a poor predictive ability for the detection of MNV with an area under the curve (AUC) of 0.515. Eyes with a PED ≥300 *μ*m had a smaller probability for the correct detection of a MNV (*p*=0.015). However, the best observed cut-off was determined at 279 *μ*m. There was no influence of age, gender, pseudophakia, BCVA, CFT, or SFCT on the detection rate of MNV. Image artifacts potentially obscuring MNV visualization were observed in 3 of 32 eyes (9.4%) with no MNV detection: 1 (3.1%) masking and motion artifact, 1 (3.1%) masking artifact alone, another motion, and blink artifact (3.1%). Image artifacts were considered as irrelevant in 12 of 32 eyes (37.5%) without MNV detection in en-face SS-OCTA, while no image artifacts were identified in 17 of 32 eyes (53.1%).

## 4. Discussion

In this prospective study, we investigated the capability of the noninvasive SS-OCTA technique for the detection and localization of treatment-naive exudative nAMD in a representative number of 158 eyes. An en-face SS-OCTA sensitivity of 79.8% as opposed to dye-based angiography could be identified similar to the retrospective data published previously (75.7%) [25]. Cross-sectional SS-OCTA revealed abnormal flow in another 23 of 32 eyes (71.9%) without MNV illustration in en-face SS-OCTA (Figure 1 B3). The superiority of combining en-face SD-OCTA and cross-sectional SD-OCTA as opposed to en-face SD-OCTA alone in patients with nAMD or PCV has been reported previously [28–30]. Thus, the overall sensitivity of the MNV detection rate in SS-OCTA increased to 94.3% as compared to dye-based angiography.

A meta-analysis including 16 eligible studies with 447 CNV eyes and 414 non-CNV eyes recently indicated a comparable diagnostic value of OCTA independently of the underlying disease (87% sensitivity) [15]. A Korean study group investigated patients with exudative nAMD in SS-OCTA and published an overall sensitivity of 80.7% [31]. Furthermore, they found a comparable sensitivity of 73.5% for type 1 MNV, 100% for type 2 MNV, and 88.9% for type 3 MNV, respectively. Corvi et al. evaluated the ability of SS-OCTA to detect MNV in eyes with atrophy compared to FA, ICGA, or OCT by utilizing a different study design [32]. A multimodal imaging setting including SS-OCTA was used to create an absolute, to which each individual imaging modality was compared. OCTA appeared to be superior to the other imaging modalities (95.2% sensitivity) in case of a coexistent macular atrophy. The study group suggested to consider OCTA as part of the multimodal imaging evaluation in eyes with atrophy. In this study, all eyes compromised by MNV were included for further comparison, regardless of the presence of atrophy. The herein presented analyzation of MNV subtypes showed excellent sensitivity for all but type 1 MNV, which represents the largest subcohort in nAMD, with a detection failure of 25.3% in en-face SS-OCTA. The detection of type 1 MNV below the RPE remains a diagnostic challenge when it comes to motion contrast sensitivity. The insufficient recognition of smaller immature pathologic vessels in the highly vascularized choriocapillaris contribute to the negative selection in OCTA. Another argument is the possible signal loss in a higher PED, a subtype of type 1 MNV also referred to as vascularized or fibrovascular PED [33, 34]. For this purpose, we analyzed the detection rate in connection with the PED height (Figure 1(b)). In general, the maximum PED height seemed to be a poor predictive marker in ROC curve influencing MNV detection by en-face SS-OCTA (AUC = 0.515), regardless of the applied segmentation process. Interestingly, nondetection was most likely in eyes with a PED above 300 *µ*m. This data enhances findings formerly proposed in our retrospective analysis, which concluded an inferior detection rate in eyes with a PED > 400 *µ*m [25]. In accordance with our data, Mrejen et al. [34] tested the sensitivity of SD-OCTA in type 1 MNV according to the corresponding PED and concluded an inferior MNV detection rate in eyes with a PED > 250 *µ*m. The study group suggested the use of manual segmentation and a multimodal imaging approach in eyes with AMD and a larger PED. Further evaluation of the 32 eyes without MNV in en-face SS-OCTA revealed relevant image artifacts in 3 eyes (9.4%). Masking artifacts due to synchysis scintillans or subretinal hemorrhage, next to motion and blink artifacts were accountable for the poor image quality in these eyes. Image artifacts in OCTA are a common phenomenon in patients with retinal and choroidal pathologies, especially in nAMD [35, 36].

The anatomical localization of all MNV subtypes according to the RPE was investigated by SS-OCTA and compared to FA (Table 1). While most of the eyes with type 1, type 3, or polypoidal type 1 MNV were detected correctly, mixed types 1 and 2 MNV and particularly type 2 MNV were identified in different layers by SS-OCTA. In type 2 MNV, 5 out of 7 eyes were detected in the sub-RPE as well as the subretinal segmentation (Figure 1(a)), while the topography was unclear in another 2 eyes. No eye revealed a neovascular network exclusively in the subretinal space as defined by type 2 MNV and as classified by both graders independently. A possible explanation is the colocalization of matured feeder vessels with explicit flow signal, which derive from the choroid through the RPE and conform a neovascular complex. Another reason might be the reflection of a subretinal membrane also visible in the choriocapillaris segmentation as projection artifacts, similar to the frequent imprint of superficial retinal vessels. To our knowledge, only one study group focused on the topography of MNV in nAMD by SS-OCTA, investigating 13 eyes with mixed types 1 and 2 MNV [37]. They were able to distinguish between type 1 and type 2 MNV components by SS-OCTA. In our study, 7 eyes (46.7%) were correctly interpreted as mixed types 1 and 2 MNV by SS-OCTA.

The limitations of this study include its single center setting without reading center approval. The detection of nonexudative MNV in the fellow eye was not investigated. Therefore, no conclusion regarding specificity of SS-OCTA could be drawn. PED measurement was defined as the maximum height of PED regardless of its reflectivity pattern. Despite that, this is the largest prospective case series comparing the sensitivity of SS-OCTA to dye-based angiography including ICGA in treatment-naive exudative nAMD. Eyes with image artifacts were analyzed to demonstrate the diagnostic capability of SS-OCTA in consecutive patients with treatment-naive nAMD in a clinical setting. Furthermore, this is the first successful attempt to bring an anatomical classification solely based on SS-OCTA into practice.

To conclude, the detection capability of SS-OCTA remains inferior to dye-based angiography in treatment-naive exudative nAMD. However, the gap narrows with combined imaging modalities of SS-OCTA. The potential to classify MNV subtypes based on its localization enhances its diagnostic value.

## Figures and Tables

**Table 1:** Demographics of patients enrolled in this study.

Mean age, years (SD)	75 (8.9)
Male, numbers (%)	52 (36.6%)
Female, numbers (%)	90 (63.4%)
BCVA in ETDRS letters, mean (range)	70 (2–95)
Snellen equivalent (range)	20/40 (1/100–20/12)

SD = standard deviation; BCVA = best corrected visual acuity; ETDRS = early treatment diabetic retinopathy study.

**Table 2:** The diagnostic sensitivity of SS-OCTA compared to MNV subtypes as classified by dye-based angiography.

Dye-based angiography		SS-OCTA		
Subtypes		En-face (%)	Cross- sectional (%)	Overall (%)
Total *n* (%)	158 (100)	126 (79.8)	23 (14.5)	149 (94.3)
Type 1 MNV	103 (65.2)	77 (74.7)	18 (17.5)	95 (92.2)
Type 2 MNV	7 (4.4)	7 (100)	0	7 (100)
Type 3 MNV	16 (10.1)	15 (93.8)	1 (6.2)	16 (100)
Mixed type 1 and 2 MNV	17 (10.8)	15 (88.2)	2 (11.8)	17 (100)
Polypoidal type 1 MNV	15 (9.5)	12 (80)	2 (13.3)	14 (93.3)

SS-OCTA = swept source-optical coherence tomography angiography; AMD = age-related macular degeneration; MNV = macular neovascularization.

**Table 3:** Topography in SS-OCTA compared to MNV subtypes as classified by dye-based angiography.

Dye-based angiography	SS-OCTA			
Subtypes	Under the RPE (%)	Above the RPE (%)	Under and above the RPE (%)	Position unclear (%)
Total *n* (%)	78 (61.9)	7 (5.5.)	35 (27.8)	6 (4.8)
Type 1 MNV	61 (79.2)	1 (1.3)	14 (18.2)	1 (1.3)
Type 2 MNV	0	0	5 (71.4)	2 (28.6)
Type 3 MNV	1 (6.7)	6 (40)	8 (53.3)	0
Mixed type 1 and 2 MNV	5 (33.3)	0	7 (46.7)	3 (20)
Polypoidal type 1 MNV	11 (91.7)	0	1 (8.3)	0

MNV = macular neovascularization; SS-OCTA = swept source-optical coherence tomography angiography; RPE = retinal pigment epithelium.

## Data Availability

Daniel Ahmed, Martin Stattin, and Siamak Ansari-Shahrezaei had full access to all the data in the study and take responsibility for the integrity of the data and the accuracy of the data analysis. The datasets used and/or analyzed to support the findings of this study are available from the corresponding author on reasonable request.
